# The monoclinic polymorph of dimethyl­arsinic acid

**DOI:** 10.1107/S1600536811025505

**Published:** 2011-07-02

**Authors:** Richard Betz, Cedric McCleland, Harold Marchand

**Affiliations:** a Nelson Mandela Metropolitan University, Summerstrand Campus, Department of Chemistry, University Way, Summerstrand, PO Box 77000, Port Elizabeth 6031, South Africa

## Abstract

The title compound, C_2_H_7_AsO_2_ or [As(CH_3_)_2_O(OH)], is an organic derivative of arsinic acid, and is also known by its trivial name cacodylic acid. In contrast to the first polymorph (triclinic, space group *P* 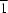, *Z* = 2), the current study revealed monoclinic symmetry (space group *C*2/*c*, *Z* = 8) for the second polymorph. The configuration of the tetra­hedral mol­ecule shows approximate *C_s_* symmetry. Strong O—H⋯O hydrogen bonds connect the mol­ecules to infinite zigzag chains along [010], which are further connected by weak inter­molecular C—H⋯O contacts into a three-dimensional network.

## Related literature

For the crystal structure of the triclinic polymorph of the title compound, see: Trotter & Zobel (1965 ▶). For graph-set analysis of hydrogen bonds, see: Etter *et al.* (1990 ▶); Bernstein *et al.* (1995 ▶).
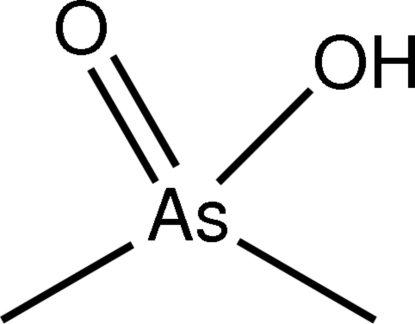


## Experimental

### 

#### Crystal data

[As(CH_3_)_2_O(OH)]
*M* *_r_* = 138.00
Monoclinic, 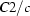

*a* = 15.764 (9) Å
*b* = 6.494 (5) Å
*c* = 11.302 (4) Å
β = 125.86 (3)°
*V* = 937.7 (10) Å^3^
*Z* = 8
Mo *K*α radiation
μ = 7.09 mm^−1^
*T* = 200 K
0.49 × 0.42 × 0.39 mm

#### Data collection

Bruker APEXII CCD diffractometer
Absorption correction: multi-scan (*SADABS*; Bruker, 2008 ▶) *T*_min_ = 0.608, *T*_max_ = 1.000
7792 measured reflections
1166 independent reflections
1117 reflections with *I* > 2σ(*I*)
*R*_int_ = 0.040

#### Refinement

*R*[*F* ^2^ > 2σ(*F* ^2^)] = 0.020
*wR*(*F* ^2^) = 0.055
*S* = 1.20
1166 reflections
49 parameters
H-atom parameters constrained
Δρ_max_ = 0.31 e Å^−3^
Δρ_min_ = −0.80 e Å^−3^

### 

Data collection: *APEX2* (Bruker, 2010 ▶); cell refinement: *SAINT* (Bruker, 2010 ▶); data reduction: *SAINT*; program(s) used to solve structure: *SHELXS97* (Sheldrick, 2008 ▶); program(s) used to refine structure: *SHELXL97* (Sheldrick, 2008 ▶); molecular graphics: *ORTEP-3* (Farrugia, 1997 ▶) and *Mercury* (Macrae *et al.*, 2008 ▶); software used to prepare material for publication: *SHELXL97* and *PLATON* (Spek, 2009 ▶).

## Supplementary Material

Crystal structure: contains datablock(s) I, global. DOI: 10.1107/S1600536811025505/wm2504sup1.cif

Supplementary material file. DOI: 10.1107/S1600536811025505/wm2504Isup2.cdx

Structure factors: contains datablock(s) I. DOI: 10.1107/S1600536811025505/wm2504Isup3.hkl

Supplementary material file. DOI: 10.1107/S1600536811025505/wm2504Isup4.cml

Additional supplementary materials:  crystallographic information; 3D view; checkCIF report

## Figures and Tables

**Table 1:** Selected bond lengths (Å)

As1—O2	1.6617 (19)
As1—O1	1.7201 (19)
As1—C2	1.895 (2)
As1—C1	1.895 (2)

**Table 2:** Hydrogen-bond geometry (Å, °)

*D*—H⋯*A*	*D*—H	H⋯*A*	*D*⋯*A*	*D*—H⋯*A*
O1—H1⋯O2^i^	0.84	1.69	2.528 (2)	172
C1—H1*A*⋯O2^ii^	0.98	2.52	3.481 (3)	167
C2—H2*B*⋯O1^iii^	0.98	2.48	3.354 (3)	148

Symmetry codes: (i) 
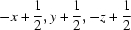
; (ii) 
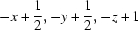
; (iii) 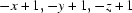.

**Fig. 1.**
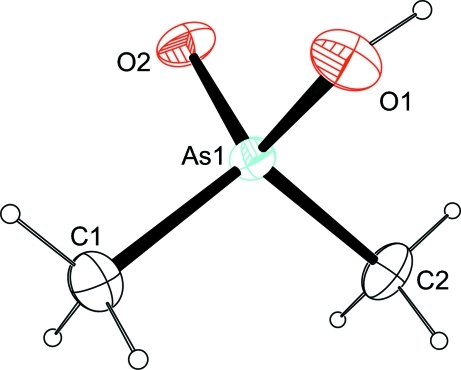
The molecular structure of the title compound, with atom labels and anisotropic displacement ellipsoids drawn at the 50% probability level.

**Fig. 2.**
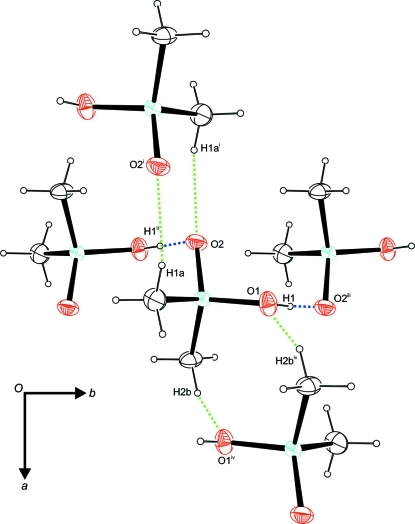
Intermolecular contacts, viewed along [001]. Blue dashed lines indicate classical O–H···O hydrogen bonds, green dashed lines C—H···O contacts. Symmetry operators: i) -x + 1/2, -y + 1/2, -z + 1; ii) -x + 1/2, y - 1/2, -z + 1/2; iii) -x + 1/2, y + 1/2, -z + 1/2; iv) -x + 1, -y + 1, -z + 1.

**Fig. 3.**
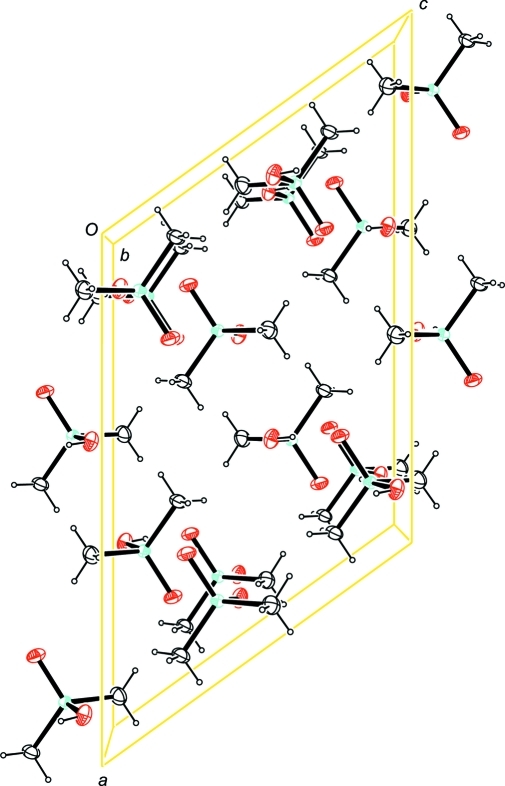
Molecular packing of the title compound, viewed along [010]; anisotropic displacement ellipsoids are drawn at the 50% probability level.

## supplementary crystallographic information

### Comment

The precipitation of amines from their respective organic synthesis mixtures as the ammonium salts of inorganic acids is a common practice for obtaining and purifying the desired products. However, for a couple of higher-alkylated amines, the viability of this class of compounds as phase-transfer catalysts becomes troublesome with respect to their persistent solubility which is detrimental for achieving quantitative yields following this simple synthetic protocol. Decreasing the solubility of a protonated amine can be done upon variation of the counterion which may allow for a better packing and more pronounced intermolecular interactions in the solid state. Since we intended to perform a comprehensive study involving a variety of higher-alkylated amines, we set out to optimize the yield of several established synthesis procedures by variation of the acid used for precipitation. To allow for a rationalization and tailoring of the counterions to be preferred, we determined the crystal structure of the title compound to enable comparative studies in isolated, crystalline precipitates. The crystal structure of the title compound has been determined previously (Trotter & Zobel (1965)). However, a different crystal system (triclinic, space group *P*1, *Z* = 2) was reported, suggesting that the title compound is polymorphic. Moreover, hydrogen atoms were not included in the previous refinement procedure.

The length of the As–O-bonds show a marked difference with the formal As–O-double bond being shorter by about 0.06 Å than the corresponding single bond. A projection of both methyl groups along the C—C-axis shows their hydrogen atoms to adopt an ecliptic conformation (Fig. 1).

In the crystal structure, O—H···O hydrogen bonds as well as intermolecular C—H···O contacts, whose range falls by about 0.2 Å below the sum of the van-der-Waals radii of the atoms participating, are present. The hydrogen bonds are formed between the H atom of the hydroxyl group as donor and the formally double-bonded oxygen atom and connect the molecules to zigzag chains along [010]. The C—H···O contacts are supported by one hydrogen atom per methyl group each as the donor atom. While for one methyl group the double bonded O atom acts as acceptor and gives rise to the formation of centrosymmetric cacodylic acid dimers, the oxygen atom of the hydroxyl group acts as acceptor for the other methyl group. In this case, too, the formation of centrosymmetric cacodylic acid dimers can be observed. In total, the molecules are connected to a three-dimensional network in the crystal structure. In terms of graph-set analysis (Etter *et al.* (1990); Bernstein *et al.* (1995)), the descriptor for the classical hydrogen bonds is *C*^1^_1_(4) on the unitary level while both C—H···O contacts necessitate a *R*^2^_2_(8) descriptor on the same level (Fig. 2).

The packing of the title compound in the crystal structure is shown in Figure 3.

### Experimental

The compound was obtained commercially (KEK). Crystals suitable for the X-ray diffraction study were taken directly from the provided product.

### Refinement

The H atoms of the methyl groups were allowed to rotate with a fixed angle around the C—As bonds to best fit the experimental electron density (HFIX 137 in the *SHELX* program suite (Sheldrick, 2008)), with *U*(H) set to 1.5*U*_eq_(C). The H atom of the hydroxyl group was found from a difference Fourier map and allowed to rotate with a fixed angle around the O—As bond to best fit the experimental electron density (HFIX 147 in the *SHELX* program suite (Sheldrick, 2008)), its *U*(H) set to 1.5*U*_eq_(O).

### Crystal data

**Table:**

[As(CH_3_)_2_O(OH)]	*F*(000) = 544
*M**_r_* = 138.00	*D*_x_ = 1.955 Mg m^−^^3^
Monoclinic, *C*2/*c*	Melting point = 468–469 K
Hall symbol: -C 2yc	Mo *K*α radiation, λ = 0.71069 Å
*a* = 15.764 (9) Å	Cell parameters from 6920 reflections
*b* = 6.494 (5) Å	θ = 2.2–28.4°
*c* = 11.302 (4) Å	µ = 7.09 mm^−^^1^
β = 125.86 (3)°	*T* = 200 K
*V* = 937.7 (10) Å^3^	Block, colourless
*Z* = 8	0.49 × 0.42 × 0.39 mm

### Data collection

**Table:**

Bruker APEXII CCD diffractometer	1166 independent reflections
Radiation source: fine-focus sealed tube	1117 reflections with *I* > 2σ(*I*)
graphite	*R*_int_ = 0.040
φ and ω scans	θ_max_ = 28.4°, θ_min_ = 3.5°
Absorption correction: multi-scan (*SADABS*; Bruker, 2008)	*h* = −20→20
*T*_min_ = 0.608, *T*_max_ = 1.000	*k* = −8→7
7792 measured reflections	*l* = −15→15

### Refinement

**Table:**

Refinement on *F*^2^	Primary atom site location: structure-invariant direct methods
Least-squares matrix: full	Secondary atom site location: difference Fourier map
*R*[*F*^2^ > 2σ(*F*^2^)] = 0.020	Hydrogen site location: inferred from neighbouring sites
*wR*(*F*^2^) = 0.055	H-atom parameters constrained
*S* = 1.20	*w* = 1/[σ^2^(*F*_o_^2^) + (0.0242*P*)^2^ + 1.2973*P*] where *P* = (*F*_o_^2^ + 2*F*_c_^2^)/3
1166 reflections	(Δ/σ)_max_ = 0.001
49 parameters	Δρ_max_ = 0.31 e Å^−^^3^
0 restraints	Δρ_min_ = −0.80 e Å^−^^3^

### Fractional atomic coordinates and isotropic or equivalent isotropic displacement parameters (Å^2^)

**Table:**

	*x*	*y*	*z*	*U*_iso_*/*U*_eq_	
As1	0.338221 (14)	0.33757 (3)	0.373791 (19)	0.01723 (9)	
O1	0.37470 (13)	0.5820 (2)	0.44526 (17)	0.0268 (3)	
H1	0.3510	0.6676	0.3770	0.040*	
O2	0.20889 (12)	0.3139 (2)	0.27582 (18)	0.0260 (3)	
C1	0.4078 (2)	0.1666 (3)	0.5414 (3)	0.0293 (5)	
H1A	0.3803	0.1934	0.5985	0.044*	
H1B	0.4830	0.1961	0.6013	0.044*	
H1C	0.3961	0.0218	0.5113	0.044*	
C2	0.38735 (18)	0.2918 (4)	0.2580 (2)	0.0270 (4)	
H2A	0.3738	0.1485	0.2243	0.041*	
H2B	0.4628	0.3188	0.3162	0.041*	
H2C	0.3509	0.3843	0.1734	0.041*	

### Atomic displacement parameters (Å^2^)

**Table:**

	*U*^11^	*U*^22^	*U*^33^	*U*^12^	*U*^13^	*U*^23^
As1	0.01664 (13)	0.01855 (13)	0.01733 (13)	0.00089 (6)	0.01041 (10)	−0.00034 (6)
O1	0.0276 (8)	0.0202 (7)	0.0229 (7)	−0.0003 (6)	0.0092 (6)	−0.0043 (6)
O2	0.0164 (7)	0.0298 (8)	0.0314 (8)	−0.0006 (6)	0.0137 (7)	−0.0059 (6)
C1	0.0327 (12)	0.0311 (12)	0.0269 (11)	0.0066 (9)	0.0189 (10)	0.0092 (8)
C2	0.0269 (11)	0.0353 (11)	0.0279 (10)	−0.0045 (9)	0.0211 (9)	−0.0053 (9)

### Geometric parameters (Å, °)

**Table:**

As1—O2	1.6617 (19)	C1—H1B	0.9800
As1—O1	1.7201 (19)	C1—H1C	0.9800
As1—C2	1.895 (2)	C2—H2A	0.9800
As1—C1	1.895 (2)	C2—H2B	0.9800
O1—H1	0.8400	C2—H2C	0.9800
C1—H1A	0.9800		
			
?···?	?		
			
O2—As1—O1	109.90 (8)	As1—C1—H1C	109.5
O2—As1—C2	111.32 (10)	H1A—C1—H1C	109.5
O1—As1—C2	108.04 (9)	H1B—C1—H1C	109.5
O2—As1—C1	112.19 (10)	As1—C2—H2A	109.5
O1—As1—C1	103.46 (10)	As1—C2—H2B	109.5
C2—As1—C1	111.56 (11)	H2A—C2—H2B	109.5
As1—O1—H1	109.5	As1—C2—H2C	109.5
As1—C1—H1A	109.5	H2A—C2—H2C	109.5
As1—C1—H1B	109.5	H2B—C2—H2C	109.5
H1A—C1—H1B	109.5		
			
?—?—?—?	?		

### Hydrogen-bond geometry (Å, °)

**Table:**

*D*—H···*A*	*D*—H	H···*A*	*D*···*A*	*D*—H···*A*
O1—H1···O2^i^	0.84	1.69	2.528 (2)	172
C1—H1A···O2^ii^	0.98	2.52	3.481 (3)	167
C2—H2B···O1^iii^	0.98	2.48	3.354 (3)	148

Symmetry codes: (i) −*x*+1/2, *y*+1/2, −*z*+1/2; (ii) −*x*+1/2, −*y*+1/2, −*z*+1; (iii) −*x*+1, −*y*+1, −*z*+1.
